# Supplementary material for: Aberrant Transferrin and Ferritin Upregulation Elicits Iron Accumulation and Oxidative Inflammaging Causing Ferroptosis and Undermines Estradiol Biosynthesis in Aging Rat Ovaries by Upregulating NF-Κb-Activated Inducible Nitric Oxide Synthase: First Demonstration of an Intricate Mechanism
Source: Int J Mol Sci. 2022 Oct 21;23(20):12689. doi: 10.3390/ijms232012689 (PMC9604315; doi:10.3390/ijms232012689)
Supplement: Supplementary file 1 [file ijms-23-12689-s001.zip › Supplementary table S4.pdf]

Supplementary table S4. Sequence distribution of biological process (Filtered by #Seqs: cutoff= 5)

GO-Terms	#Seqs	Protein
single-organism process	11	Fth1, Phb, Hspa5, Ldhb, Fabp3, Gstt3, Cbr1, Ftl1, Hba1, Tf, Selenbp2
cellular process	11	Fth1, Phb, Hspa5, Ldhb, Fabp3, Gstt3, Cbr1, Ftl1, Tf, Hba1, Selenbp2
single-organism cellular process	11	Fth1, Phb, Hspa5, Fabp3, Ldhb, Gstt3, Cbr1, Ftl1, Tf, Hba1, Selenbp2
metabolic process	9	Fth1, Phb, Hspa5, Ldhb, Fabp3, Gstt3, Cbr1, Ftl1, Tf
single-organism developmental process	9	Phb, Hspa5, Fabp3, Ldhb, Cbr1, Ftl1, Hba1, Tf, Selenbp2
developmental process	9	Phb, Hspa5, Fabp3, Ldhb, Cbr1, Ftl1, Hba1, Tf, Selenbp2
single-organism metabolic process	9	Fth1, Phb, Hspa5, Ldhb, Fabp3, Gstt3, Cbr1, Ftl1, Tf
localization	8	Fth1, Phb, Hspa5, Fabp3, Ftl1, Tf, Hba1, Selenbp2
cell differentiation	8	Phb, Hspa5, Fabp3, Cbr1, Ftl1, Tf, Hba1, Selenbp2
transport	8	Fth1, Phb, Hspa5, Fabp3, Ftl1, Tf, Hba1, Selenbp2
response to stimulus	8	Fth1, Phb, Hspa5, Fabp3, Gstt3, Cbr1, Tf, Hba1
multicellular organismal process	8	Phb, Hspa5, Fabp3, Ldhb, Cbr1, Ftl1, Hba1, Tf
biological regulation	8	Fth1, Phb, Hspa5, Fabp3, Cbr1, Ftl1, Tf, Hba1
anatomical structure development	8	Phb, Hspa5, Fabp3, Ldhb, Cbr1, Ftl1, Hba1, Tf
establishment of localization	8	Fth1, Phb, Hspa5, Fabp3, Ftl1, Tf, Hba1, Selenbp2
cellular developmental process	8	Phb, Hspa5, Fabp3, Cbr1, Ftl1, Tf, Hba1, Selenbp2
system development	7	Phb, Hspa5, Fabp3, Ldhb, Ftl1, Hba1, Tf
cellular biosynthetic process	7	Phb, Hspa5, Fabp3, Ldhb, Gstt3, Cbr1, Tf
heterocycle metabolic process	7	Phb, Hspa5, Ldhb, Fabp3, Gstt3, Cbr1, Tf
multicellular organism development	7	Phb, Hspa5, Fabp3, Ldhb, Ftl1, Hba1, Tf
biosynthetic process	7	Phb, Hspa5, Fabp3, Ldhb, Gstt3, Cbr1, Tf
response to chemical	7	Phb, Hspa5, Fabp3, Gstt3, Cbr1, Hba1, Tf
organic substance metabolic process	7	Phb, Hspa5, Ldhb, Fabp3, Gstt3, Cbr1, Tf
organic substance biosynthetic process	7	Phb, Hspa5, Fabp3, Ldhb, Gstt3, Cbr1, Tf
single-organism localization	7	Fth1, Phb, Hspa5, Fabp3, Ftl1, Hba1, Tf
single-multicellular organism process	7	Phb, Hspa5, Fabp3, Ldhb, Ftl1, Hba1, Tf
response to stress	7	Phb, Hspa5, Fabp3, Gstt3, Cbr1, Tf, Hba1
animal organ development	7	Phb, Hspa5, Fabp3, Ldhb, Ftl1, Hba1, Tf
organic cyclic compound metabolic process	7	Phb, Hspa5, Ldhb, Fabp3, Gstt3, Cbr1, Tf
nitrogen compound metabolic process	7	Phb, Hspa5, Ldhb, Fabp3, Gstt3, Cbr1, Tf
primary metabolic process	7	Phb, Hspa5, Ldhb, Fabp3, Gstt3, Cbr1, Tf
cellular metabolic process	7	Phb, Hspa5, Ldhb, Fabp3, Gstt3, Cbr1, Tf
regulation of biological quality	7	Fth1, Phb, Hspa5, Fabp3, Ftl1, Hba1, Tf

Supplementary table S4 continued. Sequence distribution of biological process (Filtered by #Seqs: cutoff= 5)

GO-Terms	#Seqs	Protein
nucleobase-containing compound metabolic process	6	Phb, Hspa5, Ldhb, Fabp3, Gstt3, Tf
cellular nitrogen compound metabolic process	6	Phb, Hspa5, Ldhb, Fabp3, Gstt3, Tf
regulation of cellular process	6	Fth1, Phb, Hspa5, Fabp3, Cbr1, Tf
regulation of biological process	6	Fth1, Phb, Hspa5, Fabp3, Cbr1, Tf
cellular aromatic compound metabolic process	6	Phb, Hspa5, Ldhb, Fabp3, Gstt3, Tf
response to organic substance	6	Phb, Hspa5, Fabp3, Cbr1, Tf, Hba1
phosphorylation	5	Phb, Hspa5, Fabp3, Ldhb, Tf
cellular response to stimulus	5	Phb, Hspa5, Fabp3, Gstt3, Tf
phosphate-containing compound metabolic process	5	Phb, Hspa5, Ldhb, Fabp3, Tf
positive regulation of cellular process	5	Fth1, Phb, Hspa5, Fabp3, Tf
phosphorus metabolic process	5	Phb, Hspa5, Ldhb, Fabp3, Tf
positive regulation of metabolic process	5	Fth1, Phb, Hspa5, Fabp3, Tf
organonitrogen compound metabolic process	5	Phb, Ldhb, Fabp3, Gstt3, Cbr1
macromolecule modification	5	Phb, Hspa5, Fabp3, Gstt3, Tf
neurological system process	5	Phb, Fabp3, Cbr1, Ftl1, Tf
organic substance catabolic process	5	Phb, Hspa5, Ldhb, Fabp3, Cbr1
cellular response to chemical stimulus	5	Phb, Hspa5, Fabp3, Gstt3, Tf
negative regulation of biological process	5	Fth1, Phb, Hspa5, Fabp3, Tf
macromolecule metabolic process	5	Phb, Hspa5, Fabp3, Gstt3, Tf
regulation of molecular function	5	Fth1, Phb, Hspa5, Fabp3, Tf
oxidation-reduction process	5	Fth1, Ldhb, Fabp3, Cbr1, Ftl1
catabolic process	5	Phb, Hspa5, Ldhb, Fabp3, Cbr1
negative regulation of cellular process	5	Fth1, Phb, Hspa5, Fabp3, Tf
cellular macromolecule metabolic process	5	Phb, Hspa5, Fabp3, Gstt3, Tf
organic cyclic compound biosynthetic process	5	Phb, Hspa5, Fabp3, Cbr1, Tf
positive regulation of biological process	5	Fth1, Phb, Hspa5, Fabp3, Tf
homeostatic process	5	Fth1, Fabp3, Ftl1, Tf, Hba1
small molecule metabolic process	5	Phb, Hspa5, Ldhb, Fabp3, Cbr1
heterocycle biosynthetic process	5	Phb, Hspa5, Fabp3, Cbr1, Tf
macromolecule localization	5	Phb, Hspa5, Fabp3, Tf, Selenbp2
regulation of metabolic process	5	Fth1, Phb, Hspa5, Fabp3, Tf
nucleic acid metabolic process	5	Phb, Hspa5, Fabp3, Gstt3, Tf
system process	5	Phb, Fabp3, Cbr1, Ftl1, Tf
cell development	5	Phb, Hspa5, Ftl1, Tf, Hba1
