# Supplementary material for: Aberrant Transferrin and Ferritin Upregulation Elicits Iron Accumulation and Oxidative Inflammaging Causing Ferroptosis and Undermines Estradiol Biosynthesis in Aging Rat Ovaries by Upregulating NF-Κb-Activated Inducible Nitric Oxide Synthase: First Demonstration of an Intricate Mechanism
Source: Int J Mol Sci. 2022 Oct 21;23(20):12689. doi: 10.3390/ijms232012689 (PMC9604315; doi:10.3390/ijms232012689)
Supplement: Supplementary file 1 [file ijms-23-12689-s001.zip › Supplementary table S5.pdf]

Supplementary table S5. Sequence distribution of cellular component (Filtered by #Seqs: cutoff= 5)

GO-Terms	#Seqs	Protein
intracellular organelle	11	Fth1, Phb, Hspa5, Fabp3, Ldhb, Gstt3, Cbr1, Ftl1, Hba1, Tf, Selenbp2
extracellular region part	11	Fth1, Phb, Hspa5, Fabp3, Ldhb, Gstt3, Cbr1, Ftl1, Tf, Hba1, Selenbp2
cell part	11	Fth1, Phb, Hspa5, Fabp3, Ldhb, Gstt3, Cbr1, Ftl1, Tf, Hba1, Selenbp2
extracellular region	11	Fth1, Phb, Hspa5, Fabp3, Ldhb, Gstt3, Cbr1, Ftl1, Tf, Hba1, Selenbp2
intracellular	11	Fth1, Phb, Hspa5, Fabp3, Ldhb, Gstt3, Cbr1, Ftl1, Tf, Hba1, Selenbp2
extracellular organelle	11	Fth1, Phb, Hspa5, Fabp3, Ldhb, Gstt3, Cbr1, Ftl1, Tf, Hba1, Selenbp2
cytoplasmic part	11	Fth1, Phb, Hspa5, Fabp3, Ldhb, Gstt3, Cbr1, Ftl1, Hba1, Tf, Selenbp2
vesicle	11	Fth1, Phb, Hspa5, Fabp3, Ldhb, Gstt3, Cbr1, Ftl1, Tf, Hba1, Selenbp2
cell	11	Fth1, Phb, Hspa5, Fabp3, Ldhb, Gstt3, Cbr1, Ftl1, Tf, Hba1, Selenbp2
membrane-bounded organelle	11	Fth1, Phb, Hspa5, Fabp3, Ldhb, Gstt3, Cbr1, Ftl1, Tf, Hba1, Selenbp2
organelle	11	Fth1, Phb, Hspa5, Fabp3, Ldhb, Gstt3, Cbr1, Ftl1, Tf, Hba1, Selenbp2
intracellular part	11	Fth1, Phb, Hspa5, Fabp3, Ldhb, Gstt3, Cbr1, Ftl1, Tf, Hba1, Selenbp2
extracellular vesicle	11	Fth1, Phb, Hspa5, Fabp3, Ldhb, Gstt3, Cbr1, Ftl1, Tf, Hba1, Selenbp2
cytoplasm	11	Fth1, Phb, Hspa5, Fabp3, Ldhb, Gstt3, Cbr1, Ftl1, Hba1, Tf, Selenbp2
extracellular exosome	11	Fth1, Phb, Hspa5, Fabp3, Ldhb, Gstt3, Cbr1, Ftl1, Tf, Hba1, Selenbp2
intracellular membrane-bounded organelle	10	Fth1, Phb, Hspa5, Fabp3, Ldhb, Gstt3, Cbr1, Ftl1, Tf, Selenbp2
cytosol	8	Fth1, Fabp3, Ldhb, Gstt3, Cbr1, Ftl1, Hba1, Selenbp2
organelle part	8	Phb, Hspa5, Fabp3, Cbr1, Ftl1, Hba1, Tf, Selenbp2
nucleus	8	Fth1, Phb, Hspa5, Fabp3, Gstt3, Cbr1, Tf, Selenbp2
membrane	8	Phb, Hspa5, Ldhb, Cbr1, Ftl1, Hba1, Tf, Selenbp2
intracellular organelle part	7	Phb, Hspa5, Fabp3, Cbr1, Hba1, Tf, Selenbp2
membrane-enclosed lumen	6	Phb, Hspa5, Fabp3, Cbr1, Tf, Selenbp2
extracellular space	6	Hspa5, Fabp3, Cbr1, Tf, Hba1, Selenbp2

Supplementary table S5 continued. Sequence distribution of cellular component (Filtered by #Seqs: cutoff= 5)

GO-Terms	#Seqs	Protein
organelle lumen	6	Phb, Hspa5, Fabp3, Cbr1, Tf, Selenbp2
cell periphery	6	Phb, Hspa5, Fabp3, Cbr1, Ftl1, Tf
intracellular organelle lumen	6	Phb, Hspa5, Fabp3, Cbr1, Tf, Selenbp2
protein complex	6	Fth1, Phb, Hspa5, Ftl1, Tf, Hba1
macromolecular complex	6	Fth1, Phb, Hspa5, Ftl1, Hba1, Tf
mitochondrion	5	Fth1, Phb, Hspa5, Ldhb, Fabp3
plasma membrane	5	Phb, Hspa5, Cbr1, Ftl1, Tf
membrane part	5	Phb, Hspa5, Ldhb, Ftl1, Tf
nuclear part	5	Phb, Hspa5, Fabp3, Cbr1, Selenbp2
non-membrane-bounded organelle	5	Phb, Hspa5, Fabp3, Hba1, Selenbp2
intracellular non-membrane-bounded organelle	5	Phb, Hspa5, Fabp3, Hba1, Selenbp2
