# Supplementary material for: Aberrant Transferrin and Ferritin Upregulation Elicits Iron Accumulation and Oxidative Inflammaging Causing Ferroptosis and Undermines Estradiol Biosynthesis in Aging Rat Ovaries by Upregulating NF-Κb-Activated Inducible Nitric Oxide Synthase: First Demonstration of an Intricate Mechanism
Source: Int J Mol Sci. 2022 Oct 21;23(20):12689. doi: 10.3390/ijms232012689 (PMC9604315; doi:10.3390/ijms232012689)
Supplement: Supplementary file 1 [file ijms-23-12689-s001.zip › Supplementary table S6.pdf]

Supplementary table S6. Sequence distribution of molecular function (Filtered by #Seqs: cutoff= 3)

GO-terms	#Seqs	Protein
binding	10	Fth1, Phb, Hspa5, Ldhb, Fabp3, Cbr1, Ftl1, Hba1, Tf, Selenbp2
protein binding	10	Fth1, Phb, Hspa5, Ldhb, Fabp3, Cbr1, Ftl1, Hba1, Tf, Selenbp2
catalytic activity	8	Fth1, Hspa5, Ldhb, Gstt3, Cbr1, Ftl1, Tf, Hba1
ion binding	7	Fth1, Hspa5, Fabp3, Cbr1, Ftl1, Tf, Hba1
heterocyclic compound binding	6	Phb, Hspa5, Ldhb, Cbr1, Tf, Hba1
organic cyclic compound binding	6	Phb, Hspa5, Ldhb, Cbr1, Tf, Hba1
small molecule binding	6	Hspa5, Fabp3, Ldhb, Cbr1, Hba1, Tf
oxidoreductase activity	6	Fth1, Ldhb, Gstt3, Cbr1, Ftl1, Hba1
metal ion binding	5	Fth1, Hspa5, Ftl1, Hba1, Tf
cation binding	5	Fth1, Hspa5, Ftl1, Tf, Hba1
anion binding	4	Hspa5, Fabp3, Cbr1, Tf
nucleoside phosphate binding	4	Hspa5, Ldhb, Cbr1, Tf
nucleotide binding	4	Hspa5, Ldhb, Cbr1, Tf
transition metal ion binding	4	Fth1, Ftl1, Tf, Hba1
iron ion binding	4	Fth1, Ftl1, Hba1, Tf
transporter activity	3	Fabp3, Hba1, Tf
ferric iron binding	3	Fth1, Ftl1, Tf
hydrolase activity	3	Hspa5, Gstt3, Tf
substrate-specific transporter activity	3	Fabp3, Tf, Hba1
enzyme binding	3	Phb, Hspa5, Ldhb
